# Targeted Therapies in the Treatment of Mantle Cell Lymphoma

**DOI:** 10.3390/cancers16101937

**Published:** 2024-05-20

**Authors:** Colin J. Thomas, Veronica Carvajal, Stefan K. Barta

**Affiliations:** 1 Department of Medicine, University of Pennsylvania, Philadelphia, PA 19104, USA; 2 Abramson Cancer Center, University of Pennsylvania, Philadelphia, PA 19104, USA

**Keywords:** mantle cell lymphoma, targeted therapy, BTK inhibitor, Bcl-2 inhibitor

## Abstract

**Simple Summary:**

Discussed here is a review of the changing landscape in the treatment of mantle cell lymphoma (MCL) regarding the emergence and use of targeted therapy. Targeted therapy is the use of small molecules designed to interrupt specific mechanisms within the cancer cell to exert anti-tumor efficacy. Targeting such mechanisms relies upon our understanding of what makes a cancer cell malignant. MCL is a difficult-to-treat lymphoid cancer that relies heavily upon constitutive intracellular signaling, promoting its growth and survival. The mainstay of MCL treatment has been to treat it with cytotoxic chemotherapy; however, targeted therapies have allowed for improved treatment outcomes and continue to change the way we manage this disease. This review aims to describe what targeted therapies are being utilized in MCL treatment and their mechanisms of action, safety, and efficacy, as well as future directions for their use in MCL treatment.

**Abstract:**

Mantle cell lymphoma (MCL) is a rare, heterogeneous B-cell non-Hodgkin’s lymphoma. The standard front-line treatment utilizes chemotherapy, often followed by consolidation with an autologous hematopoietic cell transplant; however, in most patients, the lymphoma will recur and require subsequent treatments. Additionally, mantle cell lymphoma primarily affects older patients and is frequently chemotherapy-resistant, which has further fostered the necessity for new, chemotherapy-free treatment options. In the past decade, targeted therapies in mantle cell lymphoma have been practice-changing as the treatment paradigm shifts further away from relying primarily on cytotoxic agents. Here, we will review the pathophysiology of mantle cell lymphoma and discuss the emergence of targeted, chemotherapy-free treatments aimed at disrupting the abnormal biology driving its lymphomagenesis. Treatments targeting the constitutive activation of NF-kB, Bruton’s Tyrosine Kinase signaling, and anti-apoptosis will be the primary focus as we discuss their clinical data and toxicities. Our review will also focus primarily on the emergence and use of targeted therapies in the relapsed/refractory setting but will also discuss the emergence of their use in front-line therapy and in combination with other agents.

## 1. Introduction

Mantle cell lymphoma (MCL) is a B-cell non-Hodgkin’s lymphoma (NHL), representing about 3–7% of all NHL cases in the United States [1,2]. The median age at diagnosis is approximately 68 years old, highlighting the predominantly geriatric population of patients with this disease [3]. Since MCL has traditionally been lumped into the category of the slow-growing, more indolent NHLs, it also shares a unifying characteristic within this group of malignancies: often manageable, sometimes on the scale of multiple years, yet ultimately incurable. With that said, there is heterogeneity in pathophysiology and varying degrees of how aggressive MCL may be presented.

On the spectrum of B-cell development, MCL typically originates from pre-germinal B-cells as indicated by the lack of immunoglobulin heavy-chain variable region (*IgVH*) gene somatic mutations in the most frequent, classic MCL subtype [4]. Of note, unmutated *IgVH* in chronic lymphocytic leukemia (CLL) has been associated with worse outcomes with chemotherapy-based regimens [5,6,7]. There is a non-nodal subtype of MCL, however, with the presence of *IgVH* somatic mutations and a better prognosis [8]. On the other end of the clinical spectrum, two other less frequent subtypes, blastoid and pleomorphic MCL, tend to be more aggressive with worse outcomes [9,10]. First-line treatment for MCL typically involves chemo-immunotherapy: a combination of chemotherapy with rituximab, a monoclonal antibody targeting the B-cell marker CD20 [11]. Patients with a more aggressive disease, however, have poor outcomes with upfront chemo-immunotherapy. Although some MCL patients may have favorable responses to upfront chemo-immunotherapy, producing disease control on the scale of many years [12,13,14], MCL relapse is unfortunately expected, with the disease becoming less chemo-sensitive in the relapsed/refractory (R/R) setting. Therefore, the development and implementation of chemotherapy-free treatments in MCL have been crucial. Targeted therapies represent a class of treatments that aim to disrupt specific mechanisms of pathophysiology in cancer cells, resulting in cell death without the use of cytotoxic chemotherapy. The utility of targeted therapies in MCL, therefore, hinges on our ability to understand MCL biology.

Abnormal Cylin D1 expression is a typical hallmark of MCL, usually manifested by the chromosomal 11;14 translocation, which results in the MCL cells proceeding through a steady march through the cell cycle, increasing proliferation [15]. MCL pathophysiology is also marked by deletions or mutations in the *ATM* tumor suppressor gene, noted to be present in nearly half of all new MCL cases [16,17], as well as mutations in *TP53*, noted to be present in approximately 15% of new MCL cases [18,19]. Defects in DNA repair response mechanisms further highlight the risk of chemotherapy resistance in this disease and underpin the need for chemotherapy-free approaches. Furthermore, increased Bcl-2 expression, a protein in the family of anti-apoptotic proteins, is seen in the majority of MCL cases [20]. Lastly, increased phosphoinositide 3 kinase (PI3K)/AKT signaling has been linked to promoting MCL growth; PI3K/AKT signaling is linked to B-cell receptor (BCR) signaling, Bruton’s Tyrosine Kinase (BTK) activity, and is constitutive nuclear factor-kappa B (NF-kB) activation: all resulting in B-cell growth and survival [21,22]. While no review can by any means be complete in this ever-evolving landscape, we attempted a comprehensive review of targeted agents that have published clinical data.

## 2. Targeting NF-kB

NF-kB is a family of transcription factors with an essential role in the immune system, notably in B-cell proliferation and survival [23]. Constitutive activation of this gene expression has been tied to MCL pathophysiology, which has made it an early, attractive target in treating MCL [24]. Bortezomib is a proteosome inhibitor that reversibly binds the beta subunit of the 20S proteasome, interrupting protein degradation. Preventing the degradation of IkB, a protein that maintains NF-kB in an inactivated state in the cytoplasm, is the targeted mechanism by which bortezomib primarily exerts its toxicity in MCL [25,26,27].

In the phase-two PINNACLE clinical trial, a total of 152 patients with R/R MCL were treated with bortezomib at a dose of 1.3 mg/m^2^ (intravenous [IV] or subcutaneous) on days 1, 4, 8, and 11 of a 21-day cycle for as many as 17 cycles in total [28]. The overall response rate (ORR) was 33%, and the complete response rate (CRR) was 8%. The median duration of response (DOR) was 9.2 months, with the most common grade-three or higher adverse events (AEs) being neuropathy (13%), fatigue (12%), and thrombocytopenia (11%) (Table 1). Bortezomib has also been evaluated in combination with CHOP (cyclophosphamide, doxorubicin, vincristine, and prednisone) in the R/R setting in a phase-three study [29]. A total of 46 R/R MCL patients were enrolled, half of whom received CHOP and the other half of whom received bortezomib plus CHOP. In the bortezomib–CHOP arm, bortezomib was given at a dose of 1.6 mg/m^2^ on days one and eight of a 21-day cycle for a maximum of eight cycles. The ORR with the addition of bortezomib to CHOP was 82.6% compared to 47.8% with CHOP alone; the CRR was 34.8% versus 21.7 with bortezomib–CHOP and CHOP, respectively. The median overall survival (OS) was 35.6 months with bortezomib–CHOP and 11.8 months with CHOP (*p* = 0.01, Hazard Ratio [HR]-0.37). Bortezomib–CHOP, however, was associated with an elevated risk of grade-three or higher neutropenia, 30.4%, compared to 19.6% with CHOP alone; nevertheless, grade-three or higher sensory neuropathy was similar in both arms: 6.5% with bortezomib–CHOP and 4.3% with CHOP. Bortezomib is currently FDA/EMA approved for relapsed MCL.

Given the activity of bortezomib in the R/R setting, it was studied in a front-line setting in conjunction with chemo-immunotherapy. The phase-three LYM-3002 study compared front-line R-CHOP (rituximab plus CHOP) with the regimen VR-CAP (vincristine being replaced by bortezomib) in 487 newly diagnosed MCL patients [44]. The progression-free survival (PFS) with VR-CAP versus R-CHOP was 24.7 months and 14.4 months, respectively (*p* < 0.001, HR-0.63). The CRR was 53% with VR-CAP and 42% with R-CHOP. OS at 4 years was 64% and 54% in VR-CAP and R-CHOP, respectively (*p* = 0.17, HR-0.80), underscoring the superior efficacy of VR-CAP over CHOP. VR-CAP, however, was shown to produce more significant hematologic toxicities. Grade-three or higher neutropenia was 85% in VR-CAP versus 67% with R-CHOP, while grade-three or higher thrombocytopenia was 57% with VR-CAP and 6% with R-CHOP.

Another means of targeting the NF-kB pathway in MCL is the use of lenalidomide. Lenalidomide, a chemical analog of thalidomide, has been shown to exert multiple anti-cancer mechanisms; however, it is classically considered an immunomodulatory agent due to its effect on NK cells, dendritic cells, anti-tumor T cells, and the tumor microenvironment [45]. It has been shown to inhibit the activation of IkB kinase (IKK), which, in turn, prevents the induction of genes stimulated by NF-kB [46]. Lenalidomide monotherapy was assessed in a phase-two study among 15 patients with R/R MCL who had received a median number of four prior lines of therapy [47]. Lenalidomide was given at a dose of 25 mg orally once daily on days 1–21 of a 28-day cycle for up to 52 weeks. The ORR was 53%, with a CRR of 33%. The median DOR was 13.7 months, and the median PFS was 5.6 months. The most common grade-three or higher AEs were neutropenia (40%), thrombocytopenia (33%), and leukopenia (27%) (Table 1). Lenalidomide is currently FDA/EMA approved for use in relapsed MCL.

Lenalidomide monotherapy was also assessed in a phase-two study involving a total of 26 patients with R/R MCL [30] treated at the standard dose of 25 mg orally daily on days 1–21 of a 28-day cycle for a total of six cycles. It was then followed by lenalidomide maintenance therapy in patients with a response at a dose of 15 mg daily on days 1–21 of a 28-day cycle until the progression of the disease or toxicity issues. The ORR was 31%, and the CRR was 8%. The median PFS was 14.6 months, and the DOR was 22.2 months. Lenalidomide monotherapy was also assessed in the NHL-003 study, in which it was given to a total of 57 patients with R/R MCL at a dose of 25 mg orally daily on days 1–21 of 28-day cycles until disease progression or toxicity issues [31]. The ORR was 35%, and the CRR was 12%. The median PFS was 8.8 months, and the median DOR was 16.3 months. The EMERGENCE trial studied lenalidomide monotherapy in a larger population of 134 patients with R/R MCL who had previously failed bortezomib treatment [32], resulting in an ORR of 28% and a CRR of 8%. The median PFS was 4 months, and the median DOR was 16.6 months (Table 1).

Lenalidomide was compared to the investigator’s choice of therapy in a randomized phase-two study, in which 254 patients with R/R MCL received lenalidomide or a choice of rituximab, gemcitabine, fludarabine, chlorambucil, or cytarabine [48]. Lenalidomide was given at a dose of 25 mg orally daily on days 1–21 of a 28-day cycle until the progression of the disease or toxicity issues. Patients had received a median of two previous lines of therapy, and the median age of the enrolled patients was 68.5. Lenalidomide was shown to have a significantly improved PFS compared to the investigator’s choice: 8.7 months versus 5.2 months (*p* = 0.004, HR-0.61). Lenalidomide combined with rituximab has significant activity in treatment-naïve MCL. A phase-two study was performed in which 38 treatment-naïve MCL patients were enrolled to receive lenalidomide at a dose of 20 mg orally daily for days 1–21 of a 28-day cycle for a total of 12 cycles; the dose was increased to 25 mg after cycle one if there were no dose-limiting toxicities [49]. Lenalidomide was continued at a dose of 15 mg daily on days 1–21 of a 28-day cycle for up to an additional 24 cycles. Rituximab was given at a dose of 375 mg/m^2^ during weeks 1–4, 13, 21, 29, 37, and 45 for a total of nine doses. During the maintenance phase, rituximab was given every 8 weeks for up to an additional 24 cycles. The ORR was 92%, and the CRR was 64%. The 3-year PFS and OS rates were 80% and 90%, respectively. Responses were durable, resulting in a 5-year estimated PFS and OS rate of 64% and 77%, respectively [50].

## 3. BTK Inhibitors

Targeting Bruton’s Tyrosine Kinase (BTK), an integral player in stimulating B-cell growth and proliferation as part of the B-cell receptor signaling cascade, has dramatically shifted the treatment paradigm of B-cell malignancies further into the ‘chemotherapy-free’ approach. Ibrutinib, the first-in-class BTK inhibitor established in MCL, was evaluated first in the R/R setting. Ibrutinib was designed to covalently bind the cysteine residue (C481) of the active site of the ATP binding region of BTK, thereby inhibiting its kinase function [51]. The first major study in MCL was a multi-center, phase-two trial in which 111 patients with R/R MCL were given fixed continuous doses of oral ibrutinib at a dose of 560 mg daily until the progression of the disease or issues with toxicity. The median age of the patients enrolled was 68, having received a median of three prior lines of therapy [22,35]. The ORR was 67%, and the CRR was 23%, with a median DOR of 17.5 months. The most frequent AEs of the patients included diarrhea (54%), fatigue (50%), nausea (33%), and dyspnea (32%). About 6% of the patients experienced atrial fibrillation (Afib) of any grade, most of which were grades three–four. The most common grade-three or -four infectious AEs included pneumonia (8%), urinary tract infections (4%), and cellulitis (3%). Grade-three or -four bleeding events were hematuria (2%) and subdural hematoma (2%). Regarding hematologic AEs, 22% of patients experienced thrombocytopenia, 19% experienced neutropenia, and 18% experienced anemia of any grade (Table 1).

To further validate the profound efficacy of ibrutinib in R/R MCL, ibrutinib was compared to temsirolimus in the R/R setting in a multi-center, phase-three study among patients with R/R disease [36]. Temsirolimus is an inhibitor of the mTOR pathway and has been utilized in R/R MCL, given its reported ORR of 22% in these patients [52]. A total of 139 patients were randomized to receive 560 mg oral ibrutinib daily, and 141 patients were randomized to receive IV temsirolimus (175 mg on days 1, 8, and 15 of cycle one and 75 mg on days 1, 8, and 15 of the following 21-day cycles). The median age of the patients in the trial was 68, having received a median of two prior lines of therapy. The PFS among the patients receiving ibrutinib was 14.6 months vs. 6.2 months in the temsirolimus arm (*p* < 0.0001, HR-0.43). Ibrutinib also had fewer grade-three or higher AEs, and fewer people stopped the drug due to AEs. This study further solidified the BTK inhibitor as the new standard in R/R MCL. A pooled analysis of ibrutinib in R/R MCL, which included a total of 370 patients and a 3.5-year follow-up, again underscored this drug’s practice-changing efficacy in the R/R space. The ORR was 69.7%, and the CRR was 27%, with a median DOR of 21.8 months [37,38]. Of note, patients with the blastoid MCL subtype appeared to have a lower ORR (50%) compared to the non-blastoid subtype (67.8%) as well as a lower DOR (8.5 versus 18.5 months, respectively) and PFS (5.1 months versus 14.6 months, respectively).

To improve upon ibrutinib’s AE profile, second-generation BTK inhibitors, such as acalabrutinib and zanubrutinib, were developed with the underlying concept that newer generation BTK inhibitors while targeting the same cysteine residue on BTK as ibrutinib, should have more target selectivity associated with less off-target side effects [53,54,55]. Acalabrutinib was first assessed in R/R MCL as part of a phase-two trial, in which it was given at a dose of 100 mg orally, twice a day, to a total of 124 patients until progression of the disease or issues with toxicity. The median age was 68, with patients having received a median of two prior lines of therapy [39]. The ORR was 81%, and the CRR was 40%. The most frequent grade-three or higher AEs were neutropenia (10%), anemia (9%), and pneumonia (5%). The most common grade-one and -two AEs were headache (38%), diarrhea (31%), fatigue (27%), and myalgia (21%). Importantly, there were no cases of Afib and only a single case of a grade-three or higher bleeding event. Zanubrutinib was evaluated in R/R MCL as part of a phase-two trial including a total of 86 patients; the drug was given at a dose of 160 mg twice a day orally until disease progression or issues with toxicity. The median age was 60.5, with patients having received a median of two prior lines of therapy. The ORR was 84% and the CRR was 68.6%. The most frequent grade-three or higher AEs were neutropenia (19.8%) and pneumonia (9.3%). Significant bleeding events were rare (3%), and there were no reported cases of Afib [40] (Table 1). Of note, there have been no head–head comparisons of acalabrutinib, zanubrutinib, or ibrutinib in an MCL clinical trial; with that said, the strong efficacy and, namely, side-effect profiles of acalabrutinib and zanubrutinib have established these drugs as the preferred choice over the first generation ibrutinib. Furthermore, in CLL, another B-cell malignancy, both agents showed at least similar, if not superior, efficacy to ibrutinib with an improved side-effect profile, particularly regarding cardiac adverse events [56,57,58].

Given the practice-changing success of BTK inhibitors in treating R/R MCL, it was therefore of interest to establish a BTK inhibitor with efficacy in MCL following the treatment failure of ibrutinib, acalabrutinib, or zanubrutinib, all of which bind the same cysteine residue on BTK. A common acquired mutation in CLL, which confers resistance to BTK inhibitors, is C481S, in which the cysteine residue is replaced by serine, thereby interfering with the drug’s interaction with BTK [59]. The novel, non-covalent BTK inhibitor pirtobrutinib has emerged as a means of targeting BTK following the treatment failure of these classic BTK inhibitors. Because pirtobrutinib does not bind BTK at the C481 residue, the binding site of the classic BTK inhibitors, and the site of the C481S mutation [59], it is effective in targeting both mutant C481S BTK and wild-type BTK. Because of this particular mechanism of BTK inhibitor resistance best described in CLL, pirtobrutinib was assessed in R/R MCL patients previously treated with a BTK inhibitor. In the phase-one/two BRUIN study, 90 patients with R/R MCL were treated with pirtobrutinib, all of whom were previously treated with a BTK inhibitor. The ORR was 58%, and the CRR was 20%, with a median DOR of 21.6 months. Grade-three or higher AEs were hemorrhage (3.7%) and Afib (1.2%) [41]. Of note, only 3% of patients discontinued the treatment due to AEs. The study also included 14 BTK-inhibitor-naïve patients. Among these patients, the ORR was 85.7% and the CRR was 35.7% (Table 1). Pirtobrutinib was thereby established as being safe and effective in MCL following initial BTK inhibitor failure. The FDA has since approved pirtobrutinib in R/R MCL after at least two lines of systemic treatment, including a BTK inhibitor. The EMA has also approved the use of pirtobrutinib in relapsed MCL.

Given the practice-changing success of BTK inhibitors in R/R MCL treatment, their use in the front-line setting has also been assessed. The SHINE trial was a randomized phase-three trial comparing bendamustine–rituximab (BR) vs. Ibrutinib plus BR (I-BR) in front-line MCL therapy [60]. BR was given for a total of six 28-day cycles; bendamustine was given at a dose of 90 mg/m^2^ on days 1–2; rituximab was given at a dose of 375 mg/m^2^ on day one; ibrutinib was given at a dose of 560 mg orally daily until disease progression or unacceptable side effects. Maintenance rituximab was given every 8 weeks for an additional 12 doses in patients achieving a response to treatment. Enrolled patients were 65 or older with a median age of 71. The median PFS in the I-BR group was 80.6 months, and 52.9 months in the BR group (*p* = 0.01; HR-0.75); however, the OS was similar in both groups. Although a significant PFS benefit was noted with the addition of ibrutinib to BR, it remains unclear whether combination therapy of a BTK inhibitor with BR is superior to sequential treatment with a BTK inhibitor as a second-line therapy following disease progression after BR.

The initial data of the TRIANGLE trial have also recently been presented, which have shown that the use of ibrutinib in the front-line setting of MCL treatment may be an acceptable alternative to utilizing a front-line consolidative autologous hematopoietic stem cell transplant (HSCT) [61]. In this study, previously untreated advanced-stage MCL patients were treated in one of three arms: (1) three cycles of R-CHOP/R-DHAP (alternating cycles of R-CHOP on cycles two, four, and five with R-DHAP on cycles one, three, and six [rituximab with dexamethasone, high dose cytarabine and cisplatin]) followed by autologous HCT with no ibrutinib (Arm A), (2) three cycles of R-CHOP/R-DHAP with concomitant ibrutinib and 2 years of maintenance ibrutinib (Arm I), and (3) three cycles of R-CHOP/R-DHAP with concomitant ibrutinib followed by autologous HCT and then 2 years of maintenance with ibrutinib (Arm A+I). In each treatment arm, ibrutinib was given at a dose of 560 mg orally daily. Of note, all treatment arms included maintenance rituximab. After a median follow-up of 31 months, Arm A did not show superiority over Arm-I regarding failure-free survival (FFS): the 3-year FFS was 72% (Arm A) vs. 86% (Arm I) (*p* = 0.9979, HR-1.77). Additionally, Arm A+I was superior to Arm A regarding the 3-year FFS: 88% and 72%, respectively (*p* = 0.0008, HR-0.52). Importantly, there were no significant differences between grade-three or higher AEs in the arms that added ibrutinib to upfront R-CHOP/R-DHAP chemotherapy; however, there were more grade-three or higher AEs in arms utilizing the 2-year ibrutinib maintenance during that treatment period: neutropenia 44% (Arm A+I), 17% (Arm A), and 23% (Arm I); infections 25% (Arm A+I), 13% (Arm A), and 19% (Arm I). Overall survival was similar in all arms; however, the follow-up has been too short to assess the OS endpoint, and a peer-reviewed manuscript is still pending.

Although both the SHINE and TRIANGLE trials utilized ibrutinib in the front-line setting, it is important to note that ibrutinib is no longer on the U.S. market for MCL, given the improved safety profile of acalabrutinib and zanubrutinib (acalabrutinib and zanabrutinib are FDA-approved for MCL; acalabrutinib is EMA approved for MCL); therefore, data from these studies will need to be extrapolated with the safety profile of the second generation BTK inhibitors in mind. It will also be important to establish whether the efficacy of ibrutinib in the TRIANGLE study is driven primarily by the concomitant use of ibrutinib with upfront chemotherapy or the 2-year maintenance therapy with ibrutinib.

Ibrutinib without CIT has also been assessed in the front-line setting in combination with rituximab among older MCL patients [62]. In this phase-two study, 50 newly diagnosed MCL patients with a median age of 71 were enrolled; ibrutinib was given at a dose of 560 mg orally daily in 28-day cycles until disease progression or toxicity issues; rituximab was given at a dose of 375 mg/m^2^ once weekly for cycle one, followed by day one for cycles 3–8. After cycle eight, rituximab was given every 2 months for up to 2 years. The ORR was 96%, and the CRR was 71%. The 3-year median PFS and OS were 87% and 94%, respectively. Another phase-two study also assessed the combination of ibrutinib with rituximab in a front-line MCL setting [63]. A total of 50 patients with a median age of 65 were given ibrutinib and rituximab in a similar fashion as the previously mentioned phase-two study; however, ibrutinib was discontinued after 2 years of treatment in the case of sustained, undetected minimal residual disease (MRD). The ORR was 84%, and the CRR was 80%. Longer-term follow-up and survival data are still pending. Lastly, a multi-center, real-world analysis from the United Kingdom assessed front-line ibrutinib with or without rituximab in untreated MCL [64]. Of the 104 patients assessed, the ORR was 71.2%, and the CRR was 20.2%. Of note, the ORR was higher among the patients receiving ibrutinib with rituximab as opposed to just ibrutinib alone: 78.7% versus 64.9%, respectively. Of the 39 patients with high-risk disease (*TP53* mutation/deletion, p53 overexpression, blastoid/pleomorphic subtype or Ki67 > 30%), the ORR was 59%, and the CRR was 20.5%.

## 4. BCL-2 Inhibitor

B-cell lymphoma-2 (Bcl-2) is a member of the family of anti-apoptotic proteins. It is frequently relied upon in B-cell lymphomas for allowing uncontrolled growth and proliferation [42]. The Bcl-2 homology domain-3 (BH-3) on Bcl-2 is essential in Bcl-2′s function in anti-apoptotic signaling. The Bcl-2 inhibitor, venetoclax, is a BH-3 mimetic compound and is a potent inhibitor of Bcl-2 function, resulting in apoptosis of aberrant B-cell lymphoma cells [42]. Venetoclax has been most widely studied in the R/R of MCL treatment.

A phase-one study by Davids et al. assessed venetoclax in 28 R/R MCL patients, all of whom had never been treated with a prior BTK inhibitor [43]. The ORR was 75%, with a CRR of 21%. The median age of the patients enrolled was 72 years old, with patients receiving a median of three prior lines of therapy. The estimated PFS was 14 months. All MCL patients in the trial were treated in the dose-escalation cohort, reaching daily target doses of 200–1200 mg venetoclax orally until the progression of the disease or toxicity issues. The dose of 800 mg orally daily was deemed the safest dose sufficient to achieve a strong response. A retrospective study also assessed venetoclax as monotherapy among 20 patients with R/R MCL, all of whom had previously failed with a BTK inhibitor [65]. The median age of the patients in this study was 69 years, with a median number of three lines of previous treatment. The ORR was 53%, and the CRR was 18%. The median PFS in this on-BTKi-naïve population was significantly lower at 3.2 months, and the median DOR was 8.1 months (Table 1). Finally, a retrospective study assessed the use of venetoclax monotherapy among 50 patients with R/R MCL, in addition to outcomes among 16 patients with the combination of venetoclax and a BTK inhibitor [66]. Of the 67 total patients who could be assessed for a response (including venetoclax monotherapy and in combination with a BTK inihibitor), the ORR was 40%, and the CRR was 16%. The median PFS and OS were 2.8 months and 9.5 months, respectively, and slightly longer among the patients who concomitantly received a BTK inhibitor (Table 1). Of note, venetoclax is not currently FDA/EMA approved for MCL. The combination of targeted therapies will be discussed in more detail in a section to come.

## 5. PI3K Inhibitors

The increased PI3K signaling in MCL cells has also been evaluated as a means of producing a targeted therapy approach [67]. Idelalisib, an oral PI3kδ inhibitor, was assessed in a phase-one study among 40 patients with R/R MCL [33]. Idelalisib was given orally at doses ranging from 50 mg to 350 mg, either daily or twice daily, and continued for 48 weeks in the dose escalation phase and indefinitely in the cohort expansion phase. The patients enrolled had a median age of 69 and had received a median of four prior lines of therapy. The ORR was 40%, and the CRR was 5%, with a median DOR of 2.7 months and a median PFS of 3.7 months. The 1-year PFS rate was 22%. The most common grade-three or higher AEs were AST/ALT elevation (20%), diarrhea (17.5%), decreased appetite (15%), and neutropenia (10%) (Table 1).

Parsaclisib, a selective PI3kδ inhibitor, was assessed in a phase-two study in R/R MCL [34]. A total of 53 patients who were BTK-inhibitor-naïve were enrolled, in addition to 53 patients who had previously been treated with a BTK inhibitor. Parsaclisib was given at a dose of 20 mg orally for 8 weeks, followed by a dose of 20 mg orally once weekly or a dose of 2.5 mg orally daily. Among the BTK-inhibitor-naïve patients, the ORR was 70.1%, and the CRR was 15.6%, with a median DOR of 12.1 months. The cohort involving patients previously treated with a BTK inhibitor was closed early due to a lack of clinical benefit during the interim analysis. Regarding toxicity, 62% of all treated patients had a grade-three or higher AE. The most common grade-three or higher AEs included diarrhea (13.9%), neutropenia (8.3%), hypokalemia (3.7%), and colitis (3.7%) (Table 1). The lack of benefits among the patients previously treated with a BTK inhibitor poses an issue given the favorable side-effect profile and efficacy of BTK inhibitors in this disease and their use in early lines of therapy. There are currently no FDA-approved PI3K inhibitors for MCL.

## 6. Combined Targeted Therapies

Given the promise of utilizing targeted therapies in MCL in both the R/R and front-line setting, a major area of interest has been assessing regimens that combine different targeted therapies to optimize treatment efficacy. This may be particularly important for high-risk diseases that we traditionally associate with inherent chemotherapy resistance, such as TP53 abnormal MCL [19]. The combination of bortezomib and ibrutinib was assessed in a phase-one/two study involving a total of 58 patients (9 patients in the phase-one study and 49 in the phase-two component) with R/R MCL [68]. All patients were given ibrutinib and bortezomib treatment-naïve. The median age of enrolled patients was 71, and enrolled patients could not have failed more than two previous lines of therapy. Of note, nearly three-quarters of patients in this trial had at least one high-risk feature: a blastoid or pleomorphic subtype, p53 overexpression, *TP53* mutations/deletions, or Ki-67 > 30%. Bortezomib was given at a dose of 1.3 mg/m^2^ subcutaneously on days 1, 4, 8, and 11 during six 21-day cycles; ibrutinib was concomitantly given orally daily at 560 mg until disease progression or toxicity issues. The ORR was 82%, and the CRR was 22%; this increased to an ORR of 87% and a CRR of 42% during ibrutinib maintenance. The median DOR was 22.7 months, and the median PFS was 18.6 months. The most common grade-three or higher AEs included thrombocytopenia in 16.4% of patients, neutropenia in 11% of patients, lung infections in 10.9% of patients, and peripheral sensory neuropathy in 9.1% of patients (Table 2).

Combining a BTK inhibitor with venetoclax also seemed like a natural choice, given the strong efficacy and good safety profiles of both agents. The AIM study was a phase-two study that assessed the combination of ibrutinib with venetoclax among 23 patients with R/R MCL [69,70]. Ibrutinib was initiated first as monotherapy at a dose of 560 mg orally daily for weeks 1–4; venetoclax was added during week five initially at a dose of 50 mg orally daily, which proceeded to a ramp-up schedule to a final dose of 400 mg orally daily. The study protocol was later amended to allow for the dose escalation of venetoclax to 800 mg orally daily after week 16 if a complete response (CR) had not occurred. Both drugs were continued until disease progression or toxicity issues. The ORR and CRR in week 17 were 71% and 62%, respectively. Of note, nearly half of the patients enrolled had *TP53* gene mutations/deletions. The most common grade-one/two AEs included diarrhea (83%), fatigue (75%), nausea/vomiting (71%), hemorrhage (54%), and musculoskeletal pain (50%). The most common grade-three or higher AEs included neutropenia (33%), thrombocytopenia (17%), anemia (12%), diarrhea (12%), tumor lysis syndrome (8%), and Afib (8%) (Table 2).

The SYMPATICO phase-three study compared ibrutinib monotherapy with the combination of ibrutinib and venetoclax in patients with R/R MCL. A total of 134 patients received the combination of ibrutinib/venetoclax, and a total of 133 patients received ibrutinib monotherapy. The median age of the patients in the trial was 68. Ibrutinib was given until the progression of the disease or issues with toxicity, and venetoclax was given for up to 2 years; otherwise, the doses and treatment schedule were the same as described in the AIM study. The safety lead-in phase showed an ORR of 81% and a CRR of 62% with the combination regimen. Preliminary data have also shown an improvement in the median PFS with the ibrutinib/venetoclax regimen over ibrutinib, 31.9 months versus 22.1 months, respectively (*p* = 0.0052, HR-0.65) [71]. Grade-three or higher AEs included neutropenia (31%), pneumonia (13%), thrombocytopenia (13%), anemia (10%), diarrhea (8%), leukopenia (7%), and Afib (5%) (Table 2).

Furthermore, the triplet regimen of ibrutinib, venetoclax, and obinutuzumab (anti-CD20 monoclonal antibody) was assessed in the phase-one/two OASIS study, which included both treatment-naïve MCL patients and R/R patients [72]. A total of 24 patients with R/R MCL patients were assessed; ibrutinib was given at a fixed dose of 560 mg daily for at least two years until disease progression or toxicity issues. Obinutuzumab was given at a dose of 1 g IV on days 1, 8, and 15 of cycle one and then day one of only cycles 2–8 followed by every 2 months until cycle 23. Three predetermined doses of venetoclax were used in combination with venetoclax and ibrutinib: 400 mg/day, 600 mg/day, and 800 mg/day. A dose of 400 mg/day of venetoclax was chosen to complete the trial and be used in the triplet regimen. At the end of cycle two, the ORR was 84%, and the CRR was 37%; by the end of cycle six, the ORR was 71%, and the CRR was 67%. The median age of the patients was 66, and the median number of the previous lines of therapy was one. Grade-three or higher neutropenia occurred in 71% of patients, thrombocytopenia in 54% of patients, lymphopenia in 24% of patients, and hypophosphatemia in 29% of patients. Of note, this study also included a cohort of 15 treatment-naïve patients who received ibrutinib, venetoclax, and obinutuzumab. By the end of cycle two, the ORR was 93%, and the CRR was 80%; by the end of cycle six, the ORR was 93%, and the CRR was 86%. MRD testing was utilized using a polymerase chain reaction (PCR) assay targeting either the clonal immunoglobulin H (IgH) rearrangement or the t(11;14) (q13;q32) translocation (10^−5^ sensitivity). MRD negativity was achieved in 71.5% of patients with R/R disease and in 100% of patients with a treatment-naïve disease, further highlighting the depth of response achieved (Table 2).

Regarding treatment-naïve patients, another triplet regimen of acalabrutinib, venetoclax, and rituximab was evaluated in 21 MCL patents as part of a phase-one study [74]. Acalabrutinib was given at a dose of 100 mg orally twice daily until disease progression or toxicity issues; rituximab was given IV at a dose of 375 mg/m^2^ on day one of a 28-day cycle for a total of six cycles; of note, rituximab was continued as maintenance among treatment responders for up to two years. Venetoclax was started daily on cycle two with a 5-week ramp-up period to 400 mg/daily and continued for two years. The ORR was 100% and the CRR was 90%. With a median follow-up of 25.8 months, the median DOR and median PFS were not reached (Table 2). Of note, MRD testing using the clonoSEQ^®^ peripheral blood assay was utilized, which demonstrated that 14 of 16 evaluable patients for MRD achieved an MRD-negative status (10^−6^ sensitivity level) at least once during treatment.

Furthermore, the triplet regimen zanubrutinib, obinutuzumab, and venetoclax (BOVen) have been assessed in a phase-two trial involving 25 treatment-naïve MCL patients, all of whom had *TP53* mutations [73]. The median age of the patients enrolled was 65. Zanubrutinib was given at a dose of 160 mg orally twice daily, starting on day one of a 28-day cycle; obinutuzumab was given at a dose of 1000 mg on day 1 (or split on days 1–2), day 8, and day 15 of cycle one followed by day 1 of cycles 2–8; venetoclax ramp-up started on day one of cycle three to a target dose of 400 mg orally daily. At the end of cycle 24, zanubrutinib and venetoclax were able to be discontinued if MRD-negative complete remission was achieved. MRD was assessed via a peripheral blood clonoSEQ^®^ assay. The preliminary data have shown that the ORR was 95% with a CRR of 88%. The one-year PFS rate was 84%, and the OS rate was 100%. The most common AEs were low grade, including diarrhea (52%), neutropenia (28%), infusion-related reactions (24%), thrombocytopenia (20%), and rashes (20%); grade-three or higher AEs included neutropenia (12%), infusion-related reactions (8%), and COVID-19 (8%).

Finally, a phase-two study assessed the combination of lenalidomide, venetoclax, and rituximab in 28 treatment-naïve patients [75]. Lenalidomide was given at a dose of 20 mg orally daily on days 1–21 of a 28-day cycle; venetoclax was started on day eight of cycle one at a dose of 50 mg and escalated weekly to a dose of 400 mg orally daily; rituximab was given IV weekly during cycle one and then on day one on the following cycles. This triplet therapy was given for a total of up to 12 cycles. An amendment allowed patients to transition to maintenance after cycle six if in CR by imaging in conjunction with an undetectable MRD, followed by maintenance therapy in which venetoclax was continued for one year at the same 400 mg orally daily dose; lenalidomide was reduced to 10 mg and continued for two years, and rituximab was given every 2 months for a planned 36 months. The ORR was 96%, and the CRR was 86%. This study also utilized MRD testing via the clonoSEQ^®^ assay to assess the depth of response: 86% of patients achieved an MRD-negative status. The most common grade-three or higher AEs were neutropenia (75%), thrombocytopenia (61%), anemia (32%), and tumor lysis syndrome (14%). The median PFS and DOR were not reached at the median patient follow-up of 27.5 months (Table 2).

The promising efficacy of combination targeted therapies in the front-line setting is particularly appealing for patients for whom suboptimal outcomes with cytotoxic chemotherapy are expected, such as *TP53* abnormal MCL or blastoid/pleomorphic subtypes. However, this will ultimately need to be taken into consideration with the potentially practice-changing preliminary data presented from the TRIANGLE study [61] regarding optimal sequences and the combination of treatments in the MCL treatment algorithm.

## 7. Future Directions

While preliminary efficacy and safety profiles of combined targeted therapies for MCL are enticing, and data on long-term toxicities and durability are maturing, the exploration of synergies when combining such therapies with the emerging novel immunotherapies in MCL is the next frontier. For example, the TARMAC study looked to assess the combination of fixed-duration ibrutinib with chimeric antigen receptor T-cell (CAR-T) therapy (CTL019, investigational form of tisagenlecleucel) in MCL [76]. Pre-clinical data have demonstrated the added benefit of a BTK inhibitor with CAR-T in improving CAR-T efficacy; ibrutinib has been shown to improve CAR-T cell expansion in vivo and improve T-cell fitness [77,78]. The TARMAC study involved 20 patients with R/R MCL in which ibrutinib, 560 mg orally daily, was started prior to leukapheresis and continued for a minimum of 6 months after CAR-T administration (tisagenlecleucel). Patients had received a median of two lines of past therapy, half of the enrolled patients had received a prior BTK inhibitor, and half had *TP53* mutations. At 4 months post CAR-T infusion data, 80% of patients demonstrated a CR; the estimated 12-month PFS was 75%. The efficacy of the regimen was similar in patients irrespective of prior BTK inhibitor therapy or *TP53* mutation status. A total of 75% of the patients developed cytokine release syndrome (CRS); 55% of patients developed grade-one or -two CRS, and 20% experienced grade-three CRS; 10% of patients developed grade-one or -two neurotoxicity. In addition to CAR T-cell therapies, multiple bispecific antibodies are being explored in MCL.

Apart from assessing the synergy of targeted therapies with novel immunotherapies, the optimal sequencing of these will need clarification. Furthermore, other promising novel compounds in the MCL treatment space have emerged. Targeting the mucosa-associated lymphoid tissue lymphoma translocation protein 1 (MALT1), a protein essential in B-cell receptor signaling and NF-kB activation, with MALT1 inhibitors is currently under investigation [79,80]. Targeting cyclin-dependent kinase-9 (CDK9) in mantle cell lymphoma is also being explored as a means of overcoming treatment resistance in MCL [81]. Given the inherent degree of DNA repair deficiencies in MCL, poly(ADP-ribose) polymerase-1 (PARP-1) inhibitors have been thought of as promising targets in producing synthetic lethality based on PARP-1′s role in DNA repairs. Pre-clinical data have suggested modest activity of PARP-1 inhibitors in MCL with limited data on efficacy in human trials [82,83,84]. Exploring PARP-1 inhibitors further as monotherapy or in combination with other agents could be of interest. Induced myeloid leukemia cell differentiation proteins (MCL-1), another member in the family of anti-apoptotic proteins with Bcl-2, is also another attractive target in blocking anti-apoptotic signaling in MCL [85,86,87]; however, there may be safety concerns with MCL-1 inhibitors due to potential cardiotoxicity based on early trial data [88].

Receptor tyrosine kinase-like orphan receptor 1 (ROR1) is a cell-surface signaling protein associated with de-differentiated states in malignancy, as well as increased survival and proliferation [89]. Zilovertamab vedotin (ZV) is an antibody–drug conjugate (ADC) that binds ROR1 and delivers a microtubule cytotoxic payload to tumor cells. ZV was assessed in a first-in-human phase-one study in which it was given every 3 weeks until disease progression among patients with R/R hematologic malignancies. Of the 15 patients with R/R MCL, it showed an ORR of 47% and a CRR of 20% [60,90]. These early data warrant further investigation in the MCL pipeline.

Another antibody–drug conjugate, polatuzumab, has promising activity in MCL. Polatuzumab is a CD79-directed antibody coupled to a monomethyl-auristatin E (MMAE) payload with significant activity in other B-cell lymphomas. A phase-one study of polatuzumab enrolled 95 patients in separate dose-escalation cohorts for NHL and CLL plus NHL and CLL specific expansion cohorts at the recommended phase-two dose of polatuzumab with or without rituximab; this study included seven patients with R/R MCL. Four patients with R/R MCL were treated with a single-agent polatuzumab, all of whom achieved a PR (ORR 100%). Of the three patients treated with both rituximab and polatuzumab, two had a PR, and one had a stable disease (ORR 67%) [91]. When combined with the CD20xCD3 bispecific antibody Mosunetuzumab, it resulted in an ORR of 75% and a CRR of 70%. CR rates were 75% and 70% in 20 patients with R/R MCL who had received at least two prior lines of therapy [92]. Polatuzumab is currently being explored as a monotherapy and in combination with other agents in MCL in several ongoing clinical trials.

An emerging new mechanism of targeted therapy involves proteolysis-targeting chimera (PROTAC) molecules. PROTAC molecules are compounds designed to result in the degradation of target proteins in cancer cells, offering a new approach to the targeted precision of cancer treatment. The molecules bind two ligands: a target protein of interest and E3 ubiquitin ligase. The PROTAC thereby facilitates the ubiquitylation of the target protein and subsequent degradation by the proteosome [93]. PROTAC molecules aiming to degrade BTK in B-cell malignancies are making their way into clinical trials and offer a potential new tool for treating MCL [94]. Of note, early data on a first-in-human trial of a BTK degrader presented at ASH 2023 have demonstrated safety and efficacy among heavily pre-treated B-cell NHL [95]. Given the degree of DNA damage repair abnormalities in MCL, utilizing a mouse double minute 2 homolog (MDM2) degrader in MCL treatment could also be of interest as a means of boosting p53 cell-cycle arrest and apoptosis [96].

## 8. Conclusions

The emergence and use of targeted therapies in MCL treatment have changed the paradigm by which we manage this difficult disease. Targeted therapies have become the standard in an R/R setting and are making their way into front-line MCL treatment. In the years to come, it will be important to optimize their use in combination with other targeted therapies, novel immunotherapies, and chemotherapy. Determining which patients may benefit most from specific regimens will help shape our treatment algorithms. Specifically, identifying which patients may gain the most from a chemotherapy-free approach will be of the utmost importance. For this, molecular classification and the use of MRD testing to determine high-risk diseases and identify a suboptimal response early currently hold the most promise. The optimal sequence by which we use these new therapies or combination therapies will require a thorough consideration of their toxicity profiles and goals in improving overall survival. The standardization of MRD testing will also need to be established. Lastly, other novel targeted therapies in clinical development have yet to make their mark on this patient population and will need to be integrated into the treatment algorithm as more data on these treatments emerge. One thing, though, is for certain, which is that the landscape in the treatment for MCL has irreversibly changed for the better with the advent of multiple agents that can biologically target the underlying oncogenic mechanisms at play in MCL.

## Figures and Tables

**Table 1:** Targeted Monotherapy in Relapsed/Refractory Mantle Cell Lymphoma.

Drug	Study	Number of Patients	Median Lines of Therapy	ORR (CR) %	Median DOR (mos.)	Median PFS (mos.)	Most Common Grade ≥ 3 AEs	Reference
Bortezomib	Phase II	152	2	33 (8)	9.2	NR	Neuropathy (13%), Fatigue (12%), Thrombocytopenia (11%)	[28]
Lenalidomide	Phase II	26	3	31 (8)	22.2	3.9	Neutropenia (62%), Thrombocytopenia (42%), Anemia (15%)	[30]
Lenalidomide	Phase II	57	3	35 (12)	16.3	8.8	Neutropenia (46%), Thrombocytopenia (30%), Anemia (13%)	[31]
Lenalidomide	Phase II	134	3	28 (8)	16.6	4.0	Neutropenia (43%), Thrombocytopenia (28%), Anemia (11%)	[32]
								
Idelalisib	Phase I	40	4	40 (5)	2.7	3.7	Elevated AST/ALT (20%), Diarrhea (17.5%), Decreased Appetite (15%), Neutropenia (10%)	[33]
Parsaclisib	Phase II		1				Diarrhea (13.9%), Neutropenia (8.3%), Hypokalemia (3.7%), Colitis (3.7%)	[34]
BTKi pre-treated		53		30 (2)	NR	NR		
BTKi-naïve		53		70 (16)	12.1	13.6		
								
Ibrutinib	Phase II	111	3	67 (23)	17.5	13.0	Neutropenia (17%), Thrombocytopenia (13%), Anemia (11%), Hemorrhage (6%), Atrial Fibrillation (5%)	[22,35]
Ibrutinib	Phase III	139	2	72 (19)	NR	14.6	Neutropenia (13%), Thrombocytopenia (9%), Anemia (8%), Atrial Fibrillation (4%)	[36]
Ibrutinib	Meta-Analysis	370	2	70 (27)	21.8	12.5	Not reported	[37,38]
Acalabrutinib	Phase II	124	2	81 (40)	NR	NR	Neutropenia (10%), Anemia (9%), Pneumonia (5%)	[39]
Zanubrutinib	Phase II	86	2	84 (68)	19.5	22.1	Neutropenia (20%), Pneumonia/Lung Infection (9.3%)	[40]
								
Pirtobrutinib	Phase I/II		3				Infections (17.1%), Neutropenia (13.4%), Thrombocytopenia (6.7%), Hemorrhage (4%), Atrial Fibrillation/Flutter (1%)	[41]
BTKi pre-treated		90		58 (20)	21.6	7.4		
BTKi-naïve		14		86 (36)	NR	NR		
								
Venetoclax	Phase I	28	3	75 (21)	NR	14.0	Anemia (15%), Neutropenia (11%), Thrombocytopenia (9%)	[42]
Venetoclax	Retrospective	20	3	53 (18)	3.2	8.1	Pneumonia (15%), Thrombocytopenia (5%), Hemorrhage (5%), Sepsis (5%)	[43]

**Table 2:** Combined Targeted Therapy in Relapsed/Refractory and Treatment-Naïve Mantle Cell Lymphoma.

Combination Regimen	Study	Number of Patients	Treatment-Naïve	ORR (CR) %	Median DOR (mos.)	Median PFS (mos.)	Most Common Grade ≥ 3 AEs	Reference
Bortezomib, Ibrutinib	Phase I/II	58	No	82 (22)	22.7	18.6	Thrombocytopenia (16%), Neutropenia (11%), Lung Infections (11%), Neuropathy (9%)	[34]
Ibrutinib, Venetoclax	Phase II	23	No	71 (62)	NR	29.0	Neutropenia (33%), Thrombocytopenia (17%), Anemia (12%), Diarrhea (12%), Atrial Fibrillation (8%)	[68,69]
Ibrutinib, Venetoclax	Phase III	134	No	81 (62)	42.1	31.8	Neutropenia (31%), Pneumonia (13%), Thrombocytopenia (13%), Anemia (10%), Atrial Fibrillation (5%)	[70]
Ibrutinib, Venetoclax, Obinutuzumab	Phase I/II	24	No	71 (67)	NR	NR	Neutropenia (71%), Thrombocytopenia (54%), Hypophosphatemia (29%), Lymphopenia (24%)	[71]
		15	Yes	93 (86)	NR	NR		
Acalabrutinib, Venetoclax, Rituximab	Phase I	21	Yes	100 (90)	NR	NR	Infection (38.1%), Neutropenia (33.3%)	[72]
Lenalidomide, Venetoclax, Rituximab	Phase II	28	Yes	96 (86)	NR	NR	Neutropenia (75%), Thrombocytopenia (61%), Anemia (32%)	[73]
Zanubrutinib, Venetoclax, Obinutuzumab	Phase II	25	Yes	95 (88)	NR	NR	Neutropenia (12%), Infusion-Related Reaction (8%), COVID-19 (8%)	[74]

## References

[1] Armitage J.O., Weisenburger D.D. (1998). New approach to classifying non-Hodgkin’s lymphomas: Clinical features of the major histologic subtypes. Non-Hodgkin’s Lymphoma Classification Project. J. Clin. Oncol..

[2] Teras L.R., DeSantis C.E., Cerhan J.R., Morton L.M., Jemal A., Flowers C.R. (2016). 2016 US lymphoid malignancy statistics by World Health Organization subtypes. CA Cancer J. Clin..

[3] Zhou Y., Wang H., Fang W., Romaguer J.E., Zhang Y., Delasalle K.B., Kwak L., Yi Q., Du X.L., Wang M. (2008). Incidence trends of mantle cell lymphoma in the United States between 1992 and 2004. Cancer.

[4] Walsh S.H., Thorselius M., Johnson A., Soderberg O., Jerkeman M., Bjorck E., Eriksson I., Thunberg U., Landgren O., Ehinger M. (2003). Mutated VH genes and preferential VH3-21 use define new subsets of mantle cell lymphoma. Blood.

[5] Damle R.N., Wasil T., Fais F., Ghiotto F., Valetto A., Allen S.L., Buchbinder A., Budman D., Dittmar K., Kolitz J. (1999). Ig V gene mutation status and CD38 expression as novel prognostic indicators in chronic lymphocytic leukemia. Blood.

[6] Hamblin T.J., Davis Z., Gardiner A., Oscier D.G., Stevenson F.K. (1999). Unmutated Ig V(H) genes are associated with a more aggressive form of chronic lymphocytic leukemia. Blood.

[7] Lin K.I., Tam C.S., Keating M.J., Wierda W.G., O’Brien S., Lerner S., Coombes K.R., Schlette E., Ferrajoli A., Barron L.L. (2009). Relevance of the immunoglobulin VH somatic mutation status in patients with chronic lymphocytic leukemia treated with fludarabine, cyclophosphamide, and rituximab (FCR) or related chemoimmunotherapy regimens. Blood.

[8] Orchard J., Garand R., Davis Z., Babbage G., Sahota S., Matutes E., Catovsky D., Thomas P.W., Avet-Loiseau H., Oscier D. (2003). A subset of t(11;14) lymphoma with mantle cell features displays mutated IgVH genes and includes patients with good prognosis, nonnodal disease. Blood.

[9] Swerdlow S.H., Campo E., Pileri S.A., Harris N.L., Stein H., Siebert R., Advani R., Ghielmini M., Salles G.A., Zelenetz A.D. (2016). The 2016 revision of the World Health Organization classification of lymphoid neoplasms. Blood.

[10] Gerson J.N., Handorf E., Villa D., Gerrie A.S., Chapani P., Li S., Medeiros L.J., Wang M., Cohen J.B., Churnetski M. (2023). Outcomes of patients with blastoid and pleomorphic variant mantle cell lymphoma. Blood Adv..

[11] Armitage J.O., Longo D.L. (2022). Mantle-Cell Lymphoma. N. Engl. J. Med..

[12] Hermine O., Hoster E., Walewski J., Bosly A., Stilgenbauer S., Thieblemont C., Szymczyk M., Bouabdallah R., Kneba M., Hallek M. (2016). Addition of high-dose cytarabine to immunochemotherapy before autologous stem-cell transplantation in patients aged 65 years or younger with mantle cell lymphoma (MCL Younger): A randomised, open-label, phase 3 trial of the European Mantle Cell Lymphoma Network. Lancet.

[13] Rummel M.J., Niederle N., Maschmeyer G., Banat G.A., von Grunhagen U., Losem C., Kofahl-Krause D., Heil G., Welslau M., Balser C. (2013). Bendamustine plus rituximab versus CHOP plus rituximab as first-line treatment for patients with indolent and mantle-cell lymphomas: An open-label, multicentre, randomised, phase 3 non-inferiority trial. Lancet.

[14] Flinn I.W., van der Jagt R., Kahl B., Wood P., Hawkins T., MacDonald D., Simpson D., Kolibaba K., Issa S., Chang J. (2019). First-Line Treatment of Patients with Indolent Non-Hodgkin Lymphoma or Mantle-Cell Lymphoma with Bendamustine Plus Rituximab versus R-CHOP or R-CVP: Results of the BRIGHT 5-Year Follow-Up Study. J. Clin. Oncol..

[15] Seto M., Yamamoto K., Iida S., Akao Y., Utsumi K.R., Kubonishi I., Miyoshi I., Ohtsuki T., Yawata Y., Namba M. (1992). Gene rearrangement and overexpression of PRAD1 in lymphoid malignancy with t(11;14)(q13;q32) translocation. Oncogene.

[16] Stilgenbauer S., Schaffner C., Winkler D., Ott G., Leupolt E., Bentz M., Moller P., Muller-Hermelink H.K., James M.R., Lichter P. (2000). The ATM gene in the pathogenesis of mantle-cell lymphoma. Ann. Oncol..

[17] Greiner T.C., Dasgupta C., Ho V.V., Weisenburger D.D., Smith L.M., Lynch J.C., Vose J.M., Fu K., Armitage J.O., Braziel R.M. (2006). Mutation and genomic deletion status of ataxia telangiectasia mutated (ATM) and p53 confer specific gene expression profiles in mantle cell lymphoma. Proc. Natl. Acad. Sci. USA.

[18] Greiner T.C., Moynihan M.J., Chan W.C., Lytle D.M., Pedersen A., Anderson J.R., Weisenburger D.D. (1996). p53 mutations in mantle cell lymphoma are associated with variant cytology and predict a poor prognosis. Blood.

[19] Eskelund C.W., Dahl C., Hansen J.W., Westman M., Kolstad A., Pedersen L.B., Montano-Almendras C.P., Husby S., Freiburghaus C., Ek S. (2017). TP53 mutations identify younger mantle cell lymphoma patients who do not benefit from intensive chemoimmunotherapy. Blood.

[20] Menendez P., Vargas A., Bueno C., Barrena S., Almeida J., De Santiago M., Lopez A., Roa S., San Miguel J.F., Orfao A. (2004). Quantitative analysis of bcl-2 expression in normal and leukemic human B-cell differentiation. Leukemia.

[21] Song G., Ouyang G., Bao S. (2005). The activation of Akt/PKB signaling pathway and cell survival. J. Cell. Mol. Med..

[22] Wang M.L., Rule S., Martin P., Goy A., Auer R., Kahl B.S., Jurczak W., Advani R.H., Romaguera J.E., Williams M.E. (2013). Targeting BTK with Ibrutinib in Relapsed or Refractory Mantle-Cell Lymphoma. N. Engl. J. Med..

[23] Yu H., Lin L., Zhang Z., Zhang H., Hu H. (2020). Targeting NF-κB pathway for the therapy of diseases: Mechanism and clinical study. Signal Transduct. Target. Ther..

[24] Balaji S., Ahmed M., Lorence E., Yan F., Nomie K., Wang M. (2018). NF-kappaB signaling and its relevance to the treatment of mantle cell lymphoma. J. Hematol. Oncol..

[25] Adams J. (2004). The development of proteasome inhibitors as anticancer drugs. Cancer Cell.

[26] Pham L.V., Tamayo A.T., Yoshimura L.C., Lo P., Ford R.J. (2003). Inhibition of constitutive NF-kappa B activation in mantle cell lymphoma B cells leads to induction of cell cycle arrest and apoptosis. J. Immunol..

[27] Juvekar A., Manna S., Ramaswami S., Chang T.P., Vu H.Y., Ghosh C.C., Celiker M.Y., Vancurova I. (2011). Bortezomib induces nuclear translocation of IkappaBalpha resulting in gene-specific suppression of NF-kappaB–dependent transcription and induction of apoptosis in CTCL. Mol. Cancer Res..

[28] Fisher R.I., Bernstein S.H., Kahl B.S., Djulbegovic B., Robertson M.J., de Vos S., Epner E., Krishnan A., Leonard J.P., Lonial S. (2006). Multicenter phase II study of bortezomib in patients with relapsed or refractory mantle cell lymphoma. J. Clin. Oncol..

[29] Furtado M., Johnson R., Kruger A., Turner D., Rule S. (2015). Addition of bortezomib to standard dose chop chemotherapy improves response and survival in relapsed mantle cell lymphoma. Br. J. Haematol..

[30] Eve H.E., Carey S., Richardson S.J., Heise C.C., Mamidipudi V., Shi T., Radford J.A., Auer R.L., Bullard S.H., Rule S.A. (2012). Single-agent lenalidomide in relapsed/refractory mantle cell lymphoma: Results from a UK phase II study suggest activity and possible gender differences. Br. J. Haematol..

[31] Zinzani P.L., Vose J.M., Czuczman M.S., Reeder C.B., Haioun C., Polikoff J., Tilly H., Zhang L., Prandi K., Li J. (2013). Long-term follow-up of lenalidomide in relapsed/refractory mantle cell lymphoma: Subset analysis of the NHL-003 study. Ann. Oncol..

[32] Goy A., Sinha R., Williams M.E., Kalayoglu Besisik S., Drach J., Ramchandren R., Zhang L., Cicero S., Fu T., Witzig T.E. (2013). Single-agent lenalidomide in patients with mantle-cell lymphoma who relapsed or progressed after or were refractory to bortezomib: Phase II MCL-001 (EMERGE) study. J. Clin. Oncol..

[33] Kahl B.S., Spurgeon S.E., Furman R.R., Flinn I.W., Coutre S.E., Brown J.R., Benson D.M., Byrd J.C., Peterman S., Cho Y. (2014). A phase 1 study of the PI3Kdelta inhibitor idelalisib in patients with relapsed/refractory mantle cell lymphoma (MCL). Blood.

[34] Zinzani P.L., Trneny M., Ribrag V., Zilioli V.R., Walewski J., Christensen J.H., Delwail V., Rodriguez G., Venugopal P., Coleman M. (2023). Parsaclisib, a PI3Kdelta inhibitor, in relapsed and refractory mantle cell lymphoma (CITADEL-205): A phase 2 study. EClinicalMedicine.

[35] Wang M.L., Blum K.A., Martin P., Goy A., Auer R., Kahl B.S., Jurczak W., Advani R.H., Romaguera J.E., Williams M.E. (2015). Long-term follow-up of MCL patients treated with single-agent ibrutinib: Updated safety and efficacy results. Blood.

[36] Dreyling M., Jurczak W., Jerkeman M., Silva R.S., Rusconi C., Trneny M., Offner F., Caballero D., Joao C., Witzens-Harig M. (2016). Ibrutinib versus temsirolimus in patients with relapsed or refractory mantle-cell lymphoma: An international, randomised, open-label, phase 3 study. Lancet.

[37] Rule S., Dreyling M., Goy A., Hess G., Auer R., Kahl B., Cavazos N., Liu B., Yang S., Clow F. (2017). Outcomes in 370 patients with mantle cell lymphoma treated with ibrutinib: A pooled analysis from three open-label studies. Br. J. Haematol..

[38] Rule S., Dreyling M., Goy A., Hess G., Auer R., Kahl B., Hernandez-Rivas J.A., Qi K., Deshpande S., Parisi L. (2019). Ibrutinib for the treatment of relapsed/refractory mantle cell lymphoma: Extended 3.5-year follow up from a pooled analysis. Haematologica.

[39] Wang M., Rule S., Zinzani P.L., Goy A., Casasnovas O., Smith S.D., Damaj G., Doorduijn J., Lamy T., Morschhauser F. (2018). Acalabrutinib in relapsed or refractory mantle cell lymphoma (ACE-LY-004): A single-arm, multicentre, phase 2 trial. Lancet.

[40] Song Y., Zhou K., Zou D., Zhou J., Hu J., Yang H., Zhang H., Ji J., Xu W., Jin J. (2020). Treatment of Patients with Relapsed or Refractory Mantle-Cell Lymphoma with Zanubrutinib, a Selective Inhibitor of Bruton’s Tyrosine Kinase. Clin. Cancer Res..

[41] Wang M.L., Jurczak W., Zinzani P.L., Eyre T.A., Cheah C.Y., Ujjani C.S., Koh Y., Izutsu K., Gerson J.N., Flinn I. (2023). Pirtobrutinib in Covalent Bruton Tyrosine Kinase Inhibitor Pretreated Mantle-Cell Lymphoma. J. Clin. Oncol..

[42] Adams C.M., Clark-Garvey S., Porcu P., Eischen C.M. (2018). Targeting the Bcl-2 Family in B Cell Lymphoma. Front. Oncol..

[43] Davids M.S., Roberts A.W., Seymour J.F., Pagel J.M., Kahl B.S., Wierda W.G., Puvvada S., Kipps T.J., Anderson M.A., Salem A.H. (2017). Phase I First-in-Human Study of Venetoclax in Patients with Relapsed or Refractory Non-Hodgkin Lymphoma. J. Clin. Oncol..

[44] Robak T., Huang H., Jin J., Zhu J., Liu T., Samoilova O., Pylypenko H., Verhoef G., Siritanaratkul N., Osmanov E. (2015). Bortezomib-based therapy for newly diagnosed mantle-cell lymphoma. N. Engl. J. Med..

[45] Desai M., Newberry K., Ou Z., Wang M., Zhang L. (2014). Lenalidomide in relapsed or refractory mantle cell lymphoma: Overview and perspective. Ther. Adv. Hematol..

[46] Keifer J.A., Guttridge D.C., Ashburner B.P., Baldwin A.S. (2001). Inhibition of NF-kappa B activity by thalidomide through suppression of IkappaB kinase activity. J. Biol. Chem..

[47] Habermann T.M., Lossos I.S., Justice G., Vose J.M., Wiernik P.H., McBride K., Wride K., Ervin-Haynes A., Takeshita K., Pietronigro D. (2009). Lenalidomide oral monotherapy produces a high response rate in patients with relapsed or refractory mantle cell lymphoma. Br. J. Haematol..

[48] Trneny M., Lamy T., Walewski J., Belada D., Mayer J., Radford J., Jurczak W., Morschhauser F., Alexeeva J., Rule S. (2016). Lenalidomide versus investigator’s choice in relapsed or refractory mantle cell lymphoma (MCL-002; SPRINT): A phase 2, randomised, multicentre trial. Lancet Oncol..

[49] Ruan J., Martin P., Shah B., Schuster S.J., Smith S.M., Furman R.R., Christos P., Rodriguez A., Svoboda J., Lewis J. (2015). Lenalidomide plus Rituximab as Initial Treatment for Mantle-Cell Lymphoma. N. Engl. J. Med..

[50] Ruan J., Martin P., Christos P., Cerchietti L., Tam W., Shah B., Schuster S.J., Rodriguez A., Hyman D., Calvo-Vidal M.N. (2018). Five-year follow-up of lenalidomide plus rituximab as initial treatment of mantle cell lymphoma. Blood.

[51] de Rooij M.F., Kuil A., Geest C.R., Eldering E., Chang B.Y., Buggy J.J., Pals S.T., Spaargaren M. (2012). The clinically active BTK inhibitor PCI-32765 targets B-cell receptor- and chemokine-controlled adhesion and migration in chronic lymphocytic leukemia. Blood.

[52] Hess G., Herbrecht R., Romaguera J., Verhoef G., Crump M., Gisselbrecht C., Laurell A., Offner F., Strahs A., Berkenblit A. (2009). Phase III study to evaluate temsirolimus compared with investigator’s choice therapy for the treatment of relapsed or refractory mantle cell lymphoma. J. Clin. Oncol..

[53] Covey T., Barf T., Gulrajani M., Krantz F., van Lith B., Bibikova E., van de Kar B., de Zwart E., Hamdy A., Izumi R. (2015). Abstract 2596: ACP-196: A novel covalent Bruton’s tyrosine kinase (Btk) inhibitor with improved selectivity and in vivo target coverage in chronic lymphocytic leukemia (CLL) patients. Cancer Res..

[54] Li N., Sun Z., Liu Y., Guo M., Zhang Y., Zhou D., Zhang B., Su D., Zhang S., Han J. (2015). Abstract 2597: BGB-3111 is a novel and highly selective Bruton’s tyrosine kinase (BTK) inhibitor. Cancer Res..

[55] Tam C.S., Trotman J., Opat S., Burger J.A., Cull G., Gottlieb D., Harrup R., Johnston P.B., Marlton P., Munoz J. (2019). Phase 1 study of the selective BTK inhibitor zanubrutinib in B-cell malignancies and safety and efficacy evaluation in CLL. Blood.

[56] Brown J.R., Eichhorst B., Hillmen P., Jurczak W., Kazmierczak M., Lamanna N., O’Brien S.M., Tam C.S., Qiu L., Zhou K. (2023). Zanubrutinib or Ibrutinib in Relapsed or Refractory Chronic Lymphocytic Leukemia. N. Engl. J. Med..

[57] Byrd J.C., Hillmen P., Ghia P., Kater A.P., Chanan-Khan A., Furman R.R., O’Brien S., Yenerel M.N., Illes A., Kay N. (2021). Acalabrutinib versus Ibrutinib in Previously Treated Chronic Lymphocytic Leukemia: Results of the First Randomized Phase III Trial. J. Clin. Oncol..

[58] Seymour J.F., Byrd J.C., Ghia P., Kater A.P., Chanan-Khan A., Furman R.R., O’Brien S., Brown J.R., Munir T., Mato A. (2023). Detailed safety profile of acalabrutinib vs. ibrutinib in previously treated chronic lymphocytic leukemia in the ELEVATE-RR trial. Blood.

[59] Sharma S., Galanina N., Guo A., Lee J., Kadri S., Van Slambrouck C., Long B., Wang W., Ming M., Furtado L.V. (2016). Identification of a structurally novel BTK mutation that drives ibrutinib resistance in CLL. Oncotarget.

[60] Wang M.L., Jurczak W., Jerkeman M., Trotman J., Zinzani P.L., Belada D., Boccomini C., Flinn I.W., Giri P., Goy A. (2022). Ibrutinib plus Bendamustine and Rituximab in Untreated Mantle-Cell Lymphoma. N. Engl. J. Med..

[61] Dreyling M., Doorduijn J.K., Gine E., Jerkeman M., Walewski J., Hutchings M., Mey U., Riise J., Trneny M., Vergote V.K.J. (2022). Efficacy and Safety of Ibrutinib Combined with Standard First-Line Treatment or as Substitute for Autologous Stem Cell Transplantation in Younger Patients with Mantle Cell Lymphoma: Results from the Randomized Triangle Trial By the European MCL Network. Blood.

[62] Jain P., Zhao S., Lee H.J., Hill H.A., Ok C.Y., Kanagal-Shamanna R., Hagemeister F.B., Fowler N., Fayad L., Yao Y. (2022). Ibrutinib with Rituximab in First-Line Treatment of Older Patients with Mantle Cell Lymphoma. J. Clin. Oncol..

[63] Gine E., de la Cruz F., Jimenez Ubieto A., Lopez Jimenez J., Martin Garcia-Sancho A., Terol M.J., Gonzalez Barca E., Casanova M., de la Fuente A., Marin-Niebla A. (2022). Ibrutinib in Combination with Rituximab for Indolent Clinical Forms of Mantle Cell Lymphoma (IMCL-2015): A Multicenter, Open-Label, Single-Arm, Phase II Trial. J. Clin. Oncol..

[64] Tivey A., Shotton R., Eyre T.A., Lewis D., Stanton L., Allchin R., Walter H., Miall F., Zhao R., Santarsieri A. (2024). Ibrutinib as first-line therapy for mantle cell lymphoma: A multicenter, real-world UK study. Blood Adv..

[65] Eyre T.A., Walter H.S., Iyengar S., Follows G., Cross M., Fox C.P., Hodson A., Coats J., Narat S., Morley N. (2019). Efficacy of venetoclax monotherapy in patients with relapsed, refractory mantle cell lymphoma after Bruton tyrosine kinase inhibitor therapy. Haematologica.

[66] Sawalha Y., Goyal S., Switchenko J.M., Romancik J.T., Kamdar M., Greenwell I.B., Hess B.T., Isaac K.M., Portell C.A., Mejia Garcia A. (2023). A multicenter analysis of the outcomes with venetoclax in patients with relapsed mantle cell lymphoma. Blood Adv..

[67] Thorpe L.M., Yuzugullu H., Zhao J.J. (2015). PI3K in cancer: Divergent roles of isoforms, modes of activation and therapeutic targeting. Nat. Rev. Cancer.

[68] Novak U., Fehr M., Schar S., Dreyling M., Schmidt C., Derenzini E., Zander T., Hess G., Mey U., Ferrero S. (2023). Combined therapy with ibrutinib and bortezomib followed by ibrutinib maintenance in relapsed or refractory mantle cell lymphoma and high-risk features: A phase 1/2 trial of the European MCL network (SAKK 36/13). EClinicalMedicine.

[69] Tam C.S., Anderson M.A., Pott C., Agarwal R., Handunnetti S., Hicks R.J., Burbury K., Turner G., Di Iulio J., Bressel M. (2018). Ibrutinib plus Venetoclax for the Treatment of Mantle-Cell Lymphoma. N. Engl. J. Med..

[70] Handunnetti S.M., Anderson M.A., Burbury K., Hicks R.J., Birbirsa B., Bressel M., Di Iulio J., Westerman D.A., Lade S., Agarwal R. (2019). Three Year Update of the Phase II ABT-199 (Venetoclax) and Ibrutinib in Mantle Cell Lymphoma (AIM) Study. Blood.

[71] Wang M., Jurczak W., Trněný M., Belada D., Wrobel T., Ghosh N., Keating M.-M., van Meerten T., Fernandez Alvarez R., von Keudell G. (2023). Ibrutinib Combined with Venetoclax in Patients with Relapsed/Refractory Mantle Cell Lymphoma: Primary Analysis Results from the Randomized Phase 3 Sympatico Study. Blood.

[72] Le Gouill S., Morschhauser F., Chiron D., Bouabdallah K., Cartron G., Casasnovas O., Bodet-Milin C., Ragot S., Bossard C., Nadal N. (2021). Ibrutinib, obinutuzumab, and venetoclax in relapsed and untreated patients with mantle cell lymphoma: A phase 1/2 trial. Blood.

[73] Kumar A., Soumerai J., Abramson J.S., Barnes J.A., Caron P., Chabowska M., Devlin M., Dogan A., Falchi L., Garcia R.N. (2023). A Multicenter Phase 2 Trial of Zanubrutinib, Obinutuzumab, and Venetoclax (BOVen) in Patients with Treatment-Naïve, TP53-Mutant Mantle Cell Lymphoma. Blood.

[74] Wang M., Robak T., Maddocks K.J., Phillips T., Smith S.D., Gallinson D., Calvo R., Wun C.-C., Munugalavadla V., Jurczak W. (2022). Acalabrutinib Plus Venetoclax and Rituximab in Patients with Treatment-Naïve (TN) Mantle Cell Lymphoma (MCL): 2-Year Safety and Efficacy Analysis. Blood.

[75] Phillips T.J., Bond D., Takiar R., Kump K., Kandarpa M., Boonstra P., Mayer T.L., Nachar V., Wilcox R.A., Carty S.A. (2023). Adding venetoclax to lenalidomide and rituximab is safe and effective in patients with untreated mantle cell lymphoma. Blood Adv..

[76] Minson A., Hamad N., Cheah C.Y., Tam C., Blombery P., Westerman D., Ritchie D., Morgan H., Holzwart N., Lade S. (2024). CAR T cells and time-limited ibrutinib as treatment for relapsed/refractory mantle cell lymphoma: The phase 2 TARMAC study. Blood.

[77] Ruella M., Kenderian S.S., Shestova O., Fraietta J.A., Qayyum S., Zhang Q., Maus M.V., Liu X., Nunez-Cruz S., Klichinsky M. (2016). The Addition of the BTK Inhibitor Ibrutinib to Anti-CD19 Chimeric Antigen Receptor T Cells (CART19) Improves Responses against Mantle Cell Lymphoma. Clin. Cancer Res..

[78] Gill S., Vides V., Frey N.V., Hexner E.O., Metzger S., O’Brien M., Hwang W.T., Brogdon J.L., Davis M.M., Fraietta J.A. (2022). Anti-CD19 CAR T cells in combination with ibrutinib for the treatment of chronic lymphocytic leukemia. Blood Adv..

[79] Wimberger N., Ober F., Avar G., Grau M., Xu W., Lenz G., Menden M.P., Krappmann D. (2023). Oncogene-induced MALT1 protease activity drives posttranscriptional gene expression in malignant lymphomas. Blood.

[80] Hassin O., Oren M. (2023). Drugging p53 in cancer: One protein, many targets. Nat. Rev. Drug Discov..

[81] Lee W., Jiang V.C., Zhang T.C., Yan F.F., Cai Q.S., McIntosh J., Liu Y., Wang M.L. (2023). The Selective CDK9 Inhibitor VIP152 Overcame Therapeutic Resistance in Mantle Cell Lymphoma. Blood.

[82] Williamson C.T., Kubota E., Hamill J.D., Klimowicz A., Ye R., Muzik H., Dean M., Tu L., Gilley D., Magliocco A.M. (2012). Enhanced cytotoxicity of PARP inhibition in mantle cell lymphoma harbouring mutations in both ATM and p53. EMBO Mol. Med..

[83] Pratt G., Yap C., Oldreive C., Slade D., Bishop R., Griffiths M., Dyer M.J.S., Fegan C., Oscier D., Pettitt A. (2018). A multi-centre phase I trial of the PARP inhibitor olaparib in patients with relapsed chronic lymphocytic leukaemia, T-prolymphocytic leukaemia or mantle cell lymphoma. Br. J. Haematol..

[84] Soumerai J.D., Zelenetz A.D., Moskowitz C.H., Palomba M.L., Hamlin P.A., Noy A., Straus D.J., Moskowitz A.J., Younes A., Matasar M.J. (2017). The PARP Inhibitor Veliparib Can Be Safely Added to Bendamustine and Rituximab and Has Preliminary Evidence of Activity in B-Cell Lymphoma. Clin. Cancer Res..

[85] Khoury J.D., Medeiros L.J., Rassidakis G.Z., McDonnell T.J., Abruzzo L.V., Lai R. (2003). Expression of Mcl-1 in mantle cell lymphoma is associated with high-grade morphology, a high proliferative state, and p53 overexpression. J. Pathol..

[86] Prukova D., Andera L., Nahacka Z., Karolova J., Svaton M., Klanova M., Havranek O., Soukup J., Svobodova K., Zemanova Z. (2019). Cotargeting of BCL2 with Venetoclax and MCL1 with S63845 Is Synthetically Lethal In Vivo in Relapsed Mantle Cell Lymphoma. Clin. Cancer Res..

[87] Dengler M.A., Teh C.E., Thijssen R., Gangoda L., Lan P., Herold M.J., Gray D.H., Kelly G.L., Roberts A.W., Adams J.M. (2020). Potent efficacy of MCL-1 inhibitor-based therapies in preclinical models of mantle cell lymphoma. Oncogene.

[88] Yuda J., Will C., Phillips D.C., Abraham L., Alvey C., Avigdor A., Buck W., Besenhofer L., Boghaert E., Cheng D. (2023). Selective MCL-1 inhibitor ABBV-467 is efficacious in tumor models but is associated with cardiac troponin increases in patients. Commun. Med..

[89] Kipps T.J. (2022). ROR1: An orphan becomes apparent. Blood.

[90] Jiang V.C., Liu Y., McIntosh J., Jordan A.A., Leeming A., Chen Z., Jessen K.A., Lannutti B.J., Wang M. (2020). Targeting ROR1 Using the Antibody Drug Conjugate Vls-101 in Aggressive Mantle Cell Lymphoma. Blood.

[91] Palanca-Wessels M.C., Czuczman M., Salles G., Assouline S., Sehn L.H., Flinn I., Patel M.R., Sangha R., Hagenbeek A., Advani R. (2015). Safety and activity of the anti-CD79B antibody-drug conjugate polatuzumab vedotin in relapsed or refractory B-cell non-Hodgkin lymphoma and chronic lymphocytic leukaemia: A phase 1 study. Lancet Oncol..

[92] Wang M.L., Assouline S., Kamdar M., Ghosh N., Naik S., Nakhoda S.K., Chavez J.C., Jia T., Pham S., Huw L.-Y. (2023). Fixed Duration Mosunetuzumab Plus Polatuzumab Vedotin Has Promising Efficacy and a Manageable Safety Profile in Patients with BTKi Relapsed/Refractory Mantle Cell Lymphoma: Initial Results from a Phase Ib/II Study. Blood.

[93] Bekes M., Langley D.R., Crews C.M. (2022). PROTAC targeted protein degraders: The past is prologue. Nat. Rev. Drug Discov..

[94] Dobrovolsky D., Wang E.S., Morrow S., Leahy C., Faust T., Nowak R.P., Donovan K.A., Yang G., Li Z., Fischer E.S. (2019). Bruton tyrosine kinase degradation as a therapeutic strategy for cancer. Blood.

[95] Seymour J.F., Cheah C.Y., Parrondo R., Thompson M.C., Stevens D.A., Lasica M., Wang M.L., Kumar A., Trotman J., Alwan M. (2023). First Results from a Phase 1, First-in-Human Study of the Bruton’s Tyrosine Kinase (BTK) Degrader Bgb-16673 in Patients (Pts) with Relapsed or Refractory (R/R) B-Cell Malignancies (BGB-16673-101). Blood.

[96] Marcellino B.K., Yang X., Umit Kaniskan H., Brady C., Chen H., Chen K., Qiu X., Clementelli C., Herschbein L., Li Z. (2023). An MDM2 degrader for treatment of acute leukemias. Leukemia.

